# Clinical Manifestations of an Outbreak of Monkeypox Virus in Captive Chimpanzees in Cameroon, 2016

**DOI:** 10.1093/infdis/jiad601

**Published:** 2024-03-26

**Authors:** Stephanie C. Brien, Matthew LeBreton, Jeffrey B. Doty, Matthew R. Mauldin, Clint N. Morgan, Emily G. Pieracci, Jana M. Ritter, Audrey Matheny, Bibila G. Tafon, Ubald Tamoufe, Alain D. Missoup, Julius Nwobegahay, Jean Michel Takuo, Felix Nkom, Moctar M. M. Mouiche, Jean Marc K. Feussom, Kimberly Wilkins, Abel Wade, Andrea M. McCollum

**Affiliations:** 1 Royal (Dick) School of Veterinary Studies and the Roslin Institute, Easter Bush Campus, The University of Edinburgh, Roslin, United Kingdom; 2 Ape Action Africa, Mefou Park, Cameroon; 3 Mosaic, Yaoundé, Cameroon; 4 Division of High Consequence Pathogens and Pathology, US Centers for Disease Control and Prevention, Atlanta, GA, USA; 5 Metabiota, Yaoundé, Cameroon; 6 Zoology Unit, Laboratory of Biology and Physiology of Animal Organisms, Faculty of Science, University of Douala, Cameroon; 7 Centre for Army Health Research, Yaoundé, Cameroon; 8 School of Veterinary Medicine and Sciences, University of Ngaounderé, Cameroon; 9 Cameroon Epidemiological Network for Animal Diseases, Directorate of Veterinary Services, Ministry of Livestock, Fisheries and Animal Industries, Yaoundé, Cameroon; 10 National Veterinary Laboratory, Garoua, Cameroon

**Keywords:** epidemiology, mpox, One Health, outbreak, zoonosis

## Abstract

Monkeypox virus (MPXV) is a reemerging virus of global concern. An outbreak of clade I MPXV affected 20 captive chimpanzees in Cameroon in 2016. We describe the epidemiology, virology, phylogenetics, and clinical progression of this outbreak. Clinical signs included exanthema, facial swelling, perilaryngeal swelling, and eschar. Mpox can be lethal in captive chimpanzees, with death likely resulting from respiratory complications. We advise avoiding anesthesia in animals with respiratory signs to reduce the likelihood of death. This outbreak presented a risk to animal care staff. There is a need for increased awareness and a One Health approach to preparation for outbreaks in wildlife rescue centers in primate range states where MPXV occurs. Control measures should include quarantining affected animals, limiting human contacts, surveillance of humans and animals, use of personal protective equipment, and regular decontamination of enclosures.

Monkeypox virus (MPXV) is a zoonotic orthopoxvirus (OPXV). The incidence of human cases of mpox is increasing [1–4], which is hypothesized to be due to improvements in disease detection [5], waning smallpox vaccine–derived immunity [4], shifts in ecologic and environmental factors, and altered interactions at the human-wildlife interface [6]. The disease was first recognized in Denmark in 1958 in captive Asian cynomolgus monkeys (*Macaca fascicularis*) [7] that likely acquired the infection from African monkeys while in captivity [7, 8]. MPXV has a broad host range, and while the exact reservoir species or range of species has not been established, rodents, including squirrels of the genus *Funisciurus*, are commonly implicated [9–11]. The natural life cycle is likely to be a complex interaction between the reservoir hosts and incidentally infected species such as primates, including humans [2, 12].

MPXV is divided into 2 geographically distinct clades: clade I (CI; formerly the Congo Basin clade), which appears to be more virulent and is endemic to tropical forests of Central Africa [3, 13], and clade II (CII; formerly the West African clade), which is endemic to humid lowland regions from Sierra Leone to the Southwest Region of Cameroon (clade IIa) [14, 15] and caused a multinational outbreak with extensive person-to-person transmission beginning in 2022 (clade IIb) [2]. CII MPXV has also caused outbreaks in wild chimpanzees (*Pan troglodytes*) in Côte d’Ivoire [16]. Cameroon is the only country where both clades are considered endemic [1, 17].

An outbreak of CI MPXV occurred in wild-born captive chimpanzees at a sanctuary near Mbinang in Cameroon in 2014 [18]. The sanctuary housed 72 chimpanzees, but only a single enclosure of 6 animals was affected, with a 100% attack rate and 1 death (17% case fatality rate).

In this article, we describe the clinical course, epidemiology, and management of a naturally occurring CI MPXV epizootic in a group of 23 captive chimpanzees in Mefou Park in the Centre Region of Cameroon during 2016. This outbreak was in the same region as the 2014 chimpanzee outbreak (∼400 km) and most previously recorded human CI MPXV cases in Cameroon (Figure 1A).

## METHODS

### Context and Site Description

**Figure 1.**
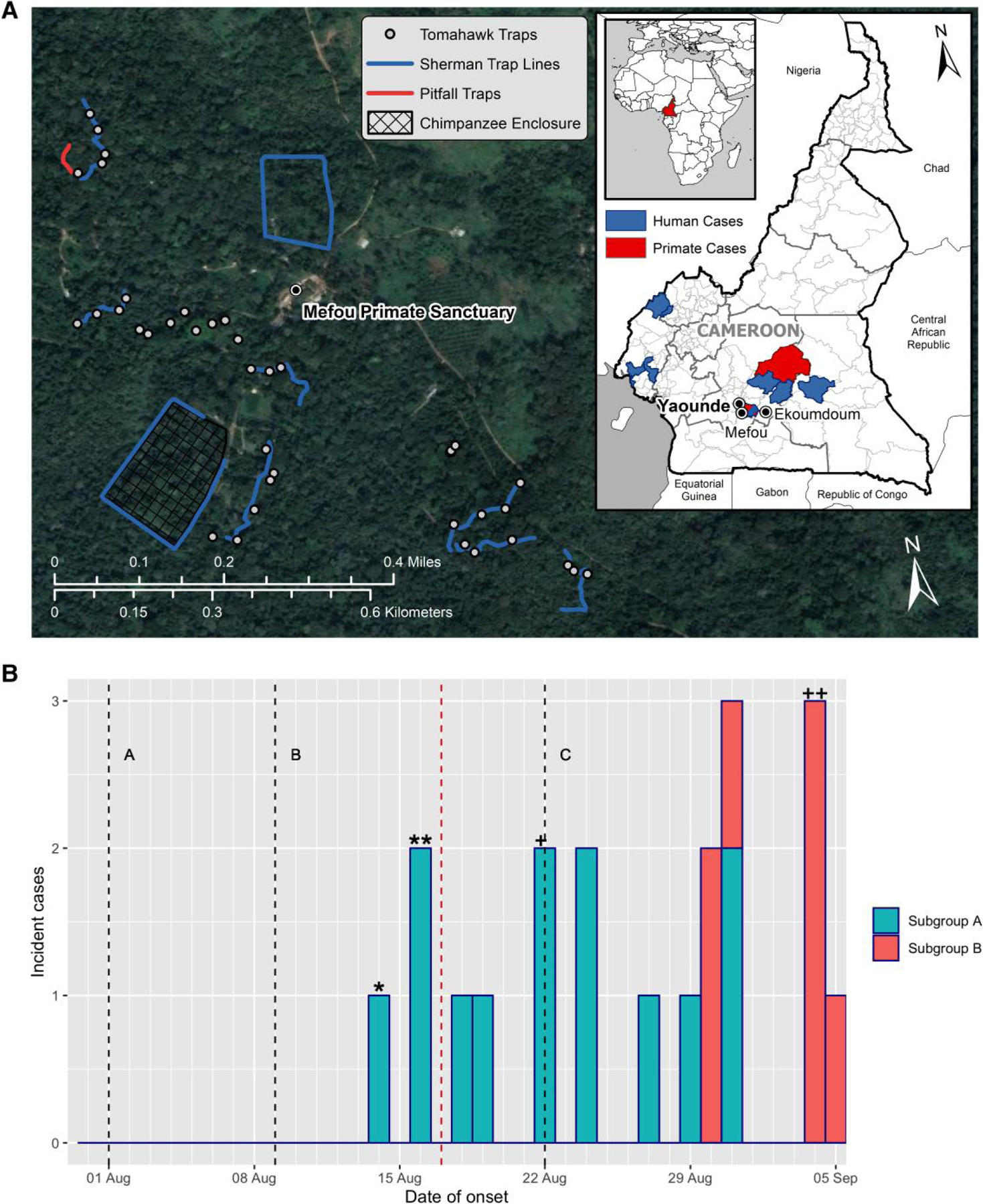
*A*, Map showing the location of Mefou Primate Sanctuary in the Centre Region of Cameroon, the affected chimpanzee enclosure, and small mammal trapping locations. For the country map (inset), Health Districts are divided by thin, light grey lines, whilst thicker, darker grey lines denote regional boundaries. Health Districts in Cameroon where mpox cases have been reported in humans and NHPs up to the end of 2021 [1, 18–20] are shaded. Single human cases of CI MPXV have been documented in the Centre region of Cameroon in 1979 [21] and 1989 [22], with more widespread outbreaks recorded since 2019 [1, 17, 19, 20]. Cases of CII MPXV were reported in the Southwest and Northwest Regions of Cameroon in 2018 and 2022–23 [1, 15, 17, 19]. The virus circulating in 2018 was closely related to strains circulating in neighboring Nigeria [15]. The primate enclosures typically comprise a covered night shelter connected to an open-air, forested area (Supplementary Figure 1). The sanctuary is within the 1044ha proposed Mefou National Park which is a mixture of intact and disturbed forest. Adjoining farmlands are cultivated on a rotating basis, with forest allowed to regenerate during the fallow period. The animals at the sanctuary were typically removed from the wild by poachers as infants and arrived in the sanctuary following confiscation or voluntary handovers from hunters, traders or people keeping them as pets. *B*, Epidemic curve shows incident cases of mpox by subgroup against date of onset. Subgroup A moved between a daytime forested enclosure and a caged night shelter, but subgroup B were confined to cages throughout (Supplementary Figure 1). Likely exposure dates and latest onset date for a point source outbreak were calculated by extrapolation of the IQR for human incubation periods for clade I mpox to the onset date in the index case. Labelled dashed lines: A = earliest likely exposure date, B = latest likely exposure date, C = latest expected onset date from a point source exposure. Unlabelled dashed line (17 Aug): last date with percutaneous direct contact between the subgroups. Index case: *date of onset, **death. Other fatality: +date of onset, +death.

The outbreak occurred from August to October 2016 at Mefou Primate Sanctuary, 25 km south of Yaoundé in the Centre Region of Cameroon (Figure 1A). At the time of the outbreak, the sanctuary housed about 300 nonhuman primates (NHPs), comprising chimpanzees, gorillas, and monkeys, divided into 18 groups. The outbreak was confined to 1 group of chimpanzees. The affected group was composed of 23 adult and subadult chimpanzees (14 males and 9 females) in good general health. There had been no movement of animals into or out of the group in the preceding 6 months. The affected group consisted of 2 subgroups (A and B) that did not mix but had the opportunity for direct contact through the bars of adjacent cages (Supplementary Figure 1): subgroup A moved between a daytime forested enclosure and a caged night shelter, whereas subgroup B was confined to cages throughout.

### Case Definitions

Confirmed cases were defined as animals with illness clinically compatible with mpox and with MPXV DNA signatures amplified by real-time polymerase chain reaction (PCR) from a diagnostic specimen. For health and safety reasons, diagnostic specimens were collected only from animals that died or required anesthesia for clinical reasons. Probable cases were defined as animals that had a clinically compatible illness characterized by (1) a maculopapular, vesicular, or pustular rash or at least 2 other compatible signs (coryza, lethargy, anorexia, cough, facial or perilaryngeal swelling) and (2) the opportunity for direct contact with a laboratory-confirmed case or the bodily fluids of a confirmed case between August and October 2016. Symptomatic animals were considered noninfectious for MPXV once all visible lesions had desquamated and fresh skin had formed.

### Clinical Evaluation and Treatment

Following the detection of the first case on 14 August 2016, the Cameroon Epidemiological Network for Animal Diseases was notified, and clinical data—including signs, date of onset, date of death, movements between cages, and treatment— were recorded daily for the affected group by the on-site veterinarian. For the 17 unaffected groups, caregivers performed a daily visual inspection of each animal for signs of illness, behavioral changes, and night shelter use. Additionally, a visual veterinary inspection of animals in these groups was performed at least twice weekly to check for signs of mpox. Animals were quarantined if they met either of the following criteria: (1) clinical signs compatible with mpox or (2) direct or indirect contact with the affected group.

### Diagnostic, Environmental, and Small Mammal Sample Collection

Oropharyngeal and rectal swabs were collected from deceased chimpanzees and chimpanzees immobilized for treatment and stored in cryotubes with or without TRIzol viral transport medium for laboratory confirmation of MPXV. A full postmortem examination was conducted on the first fatality. Gross pathologic findings were recorded, and tissues—including skin lesions, larynx, lung, and lymph node—were fixed in 10% neutral buffered formalin for at least 48 hours, transferred to 70% ethanol, and processed by routine paraffin histology at the US Centers for Disease Control and Prevention (CDC). Sections (4 µm) were mounted on glass slides and stained with hematoxylin-eosin or by immunohistochemistry via a multistep immunoalkaline phosphatase technique with a rabbit polyclonal anti-MPXV antibody, as previously described [23]. Excisional biopsies of orofacial papules were collected in viral transport medium, along with oropharyngeal and rectal swabs, and frozen at −80 °C prior to analysis at CRESAR (Military Health Research Centre) in Yaoundé.

On 26 August, samples were collected in and around the enclosure, prior to cleaning. Paired swabs were collected by rubbing a ∼10-cm linear surface for 5 seconds with a sterile Dacron-tipped swab. Small fragments (0.1 mL) of discarded bedding, food material, and feces were also collected (Supplementary Table 1). Each sample was collected in duplicate and then stored in 500 µL of TRIzol or a sterile cryotube with no medium. Samples were initially kept in a cooler with icepacks and transferred to a −80 °C freezer at CRESAR within 3 hours of collection.

In August 2016 and December 2017, small mammals were trapped and sampled in different areas across the sanctuary (Figure 1A), including locations adjacent to the affected group’s enclosure. Sherman live traps, Tomahawk live traps, and pitfall traps were deployed as previously described [24]. In 2016, animals were captured, and oral and anal swabs were collected in duplicate and then stored in 500 µL of TRIzol and 500 µL of viral transport medium. The animals were released at the site of capture. In 2017, captured small mammals were processed, euthanized, and sampled as previously described [24, 25]. This work followed approved Institutional Animal Care and Use Committee protocols (2016: UC Davis 17803, 2017: CDC 2660DOTMULX).

Transport of specimens obtained from the chimpanzees to the United States followed relevant international regulations, including those of the World Health Organization, World Organisation for Animal Health, and the Convention on International Trade in Endangered Species (export permit 200PRBS/MINFOF/SG/DFAP/SDVEF/SC and import permit 17US14106C/9).

### Laboratory Analyses

Small mammal samples collected in 2017 were processed and extracted as previously described [25]. The same procedures were used for chimpanzee and environmental samples, except that DNA was extracted with the QIAamp DNA Mini Kit and EZ1 Advanced XL Robot (Qiagen) and environmental samples were homogenized in 300 µL of phosphate-buffered saline instead of the 500 µL used for animal samples. For small mammal swabs collected in 2016, DNA was extracted from 200 µL of TRIzol supernatant via the Viral DNA Kit (Zymo Research).

Environmental samples, skin lesions, and animal swabs collected in 2016 were initially tested in Cameroon by a conventional PCR capable of identifying all members of the genus *Orthopoxvirus*. MPXV was confirmed by sequencing. Positive samples were then sent to the CDC, along with additional postmortem tissue samples and small mammal samples collected in 2017, and tested with a generic OPXV real-time PCR and a CI MPXV–specific assay [26, 27]. Samples positive for MPXV DNA were added to cell culture for viral isolation and titration as previously described [25]. Small mammal serum and blood samples collected in 2017 were assessed by modified enzyme-linked immunosorbent assay (ELISA) for the presence of anti-OPXV IgG antibodies, as previously described [24].

The MPXV genome was sequenced (from the index case’s lesion specimen) with the Illumina MiSeq benchtop system and assembled into 3 contigs through SPAdes (version 3.10.0). All contigs had coverage exceeding 1000×. Contigs were joined by manual extension with reads. In addition to the newly generated whole genome sequence, 20 CI MPXV and 11 CII MPXV reference genomes were selected as ingroup taxa, with cowpox virus strain Grisham (X94355) serving as the outgroup taxon. Genomes were aligned by the FFT-NS-2 algorithm within MAFFT (version 7.215) [28]. Phylogenetic analyses were conducted with RAxML (version 7.3.0) [29] with a GTR+I+G model of molecular evolution, 200 rapid bootstrap replicates, followed by a search for the best-scoring maximum likelihood tree within a single run.

## RESULTS

### Index Case

On the evening of 14 August 2016, the index case, a 10-year-old female in subgroup A, uncharacteristically refused to enter the night shelter from the forested enclosure. Visual inspection was unremarkable until 15 August, when dysphagia, coryza, and mild stridor became apparent. By 16 August, lethargy, dyspnea, and maculopapular exanthema had developed, and stridor was more pronounced (Supplementary Video 1). She was favoring a prone or sitting position with her head hunched over. On 16 August, the index case was anesthetized to obtain diagnostic samples, and she died under anesthesia as a result of asphyxition secondary to laryngeal edema. DNA signatures specific for CI MPXV were amplified via real-time PCR from 2 lesion specimens.

### Epidemiology

Between 14 August and 5 September 2016, 3 confirmed and 17 probable cases (87% attack rate) were detected. There were 2 fatalities (case fatality rate, 10% [2/20]). The epidemic curve (Figure 1B) illustrates that the outbreak commenced in subgroup A, with no cases in subgroup B for 16 days. From 17 August (13 days prior to onset in subgroup B), direct contact between the subgroups was prevented by leaving an empty cage between them (cage B1 in Supplementary Figure 1). The 3 clinically unaffected animals were all in subgroup A.

In humans, CI MPXV has a mean incubation period of 8 days (IQR, 5–13) [30]. This is similar to figures found in respiratory challenge studies in NHPs [31]. Based on these values, 13 cases in this outbreak (65%) occurred after 22 August, the latest expected onset date for a point source outbreak (Figure 1B, line C), suggesting chimp-to-chimp transmission.

During the outbreak, the group was mainly confined to the cages to facilitate monitoring, veterinary treatment, and containment. Animals were occasionally released into the forested enclosure to enable the cages to be cleaned. From 10 October, daily release of animals from subgroup A into the forested enclosure resumed. One female refused to enter the cages and remained in the forested enclosure throughout the outbreak. She did not develop signs of mpox.

The animals were free from exanthema and therefore no longer considered infectious from 24 October 2016.

### Clinical Presentation

The frequency and duration of clinical signs are provided in Table 1 (for additional details see supplementary materials, for photographs see Supplementary Figure 2). The most common signs were exanthema (18/20, 90%), lethargy (17/20, 85%), and facial or perilaryngeal edema (14/20, 70%). Exanthema was defined as macular, papular, or pustular skin lesions. Lesion density varied among animals, from single or sparsely distributed lesions to localized constellations or diffuse rash. At initial presentation, most animals showed skin lesions (15/20, 75%), usually accompanied by systemic signs (eg, lethargy, inappetence, behavioral change).

Perilaryngeal swelling was seen in 8 animals (40%) and was bilaterally symmetrical and often accompanied by dysphagia (7/8, 88%), dyspnea (5/8, 63%), and/or coryza (5/8, 63%). Blepharospasm and purulent ocular discharge were seen in 3 animals (15%). Two animals showed corneal clouding and developed chronic unilateral ocular sequelae associated with apparent discomfort. One of these two went on to develop corneal scarring leading to suspected unilateral blindness.

Fatalities occurred in 2 animals in subgroup A with severe exanthema, enanthema, marked coryza, and perilaryngeal swelling leading to marked dysphagia. Both animals were dyspneic with respiratory noises (stridor or wheezing), open mouth breathing, and a preference for a prone or sitting position in the 24 hours preceding death. The second fatality occurred overnight in a 9-year-old female after 13 days of illness, including lethargy (12 days) and perilaryngeal swelling (9 days). She developed a cough 4 days prior to death. She maintained the desire to eat but had difficulty swallowing, with apparent pain. She showed poor compliance with taking oral medications. Death was probably due to respiratory complications, most likely asphyxiation due to laryngeal obstruction, bronchopneumonia, or a combination of the two.

Respiratory signs were seen only in subgroup A, while inappetence, diarrhea, and weight loss were seen only in subgroup B (for further details, see supplementary materials, including Supplementary Table 2).

### Pathology and Laboratory Findings

Necropsy of the index case showed extensive maculopapular exanthema of the face, enanthema of the oral cavity, severe laryngeal swelling, and eschars on the dorsum (Supplementary Figure 2). Histology (Supplementary Figure 3) revealed necrotizing laryngitis with extensive ulceration, mixed inflammation, and fibrinosuppurative surface exudates with bacteria. Rare intracytoplasmic eosinophilic inclusions were seen in keratinocytes. At ulcer margins, there was keratinocyte degeneration, subbasilar edema and clefting, and hyperplasia of adjacent epithelium. Lung sections showed patchy bronchopneumonia, and the colon showed multiple foci of mucosal necrosis, centered on mucosa-associated lymphoid tissue in some areas.

Necrotic foci in the larynx, lung, and colon showed extensive immunohistochemical staining for MPXV. Sections of lymph node showed a mild increase in medullary and subcapsular histiocytes, which displayed scattered immunohistochemical staining. Twelve tissue samples tested from the index case were positive by a CI MPXV–specific PCR, and 9 of 12 contained detectable levels of viable MPXV on viral culture (Supplementary Table 3). MPXV-specific DNA signatures were also amplified from swabs from the second deceased animal (2/2 oral, 2/2 rectal) and a third symptomatic animal (2/2 oral, 0/2 rectal).

Phylogenetic analyses of whole genome sequence recovered reciprocally monophyletic clades of genomes from CI and CII (Supplementary Figure 4). The genome generated from the index case (196 728 base pairs, accession OR943698) formed a monophyletic group with high statistical support with a genome isolated from a child in Ékoumdoma, Cameroon (KJ642618), in 1989 [22] and a genome from Gabon in 1988 (KJ642619). The relationship of these genomes to other CI MPXV genomes is less certain, with bootstrap support varying at deeper nodes. The 2 Cameroon whole genome sequences that were examined, separated by nearly 30 years, shared 99.7% sequence identity across homologous bases with gaps removed. The localities of Ékoumdouma and Mefou, where these isolates were derived, are directly separated by ∼70 or ∼110 km via road.

### Environmental Sample Analysis

MPXV DNA signatures were amplified by PCR in 6 of 13 environmental samples collected from cages housing infected animals (Supplementary Table 3). Of these 6 samples, only 2 contained a detectable level of viable virus, although 2 were cytotoxic in cell culture and could not be titrated.

### Small Mammal Sample Analysis

The animals sampled in 2016 (n = 77; Supplementary Table 4) and 2017 (n = 85; Supplementary Table 5) belonged to 2 orders, Rodentia (n = 136, 84%) and Eulipotyphia (n = 26, 16%), and represented 11 genera. Twenty-one were not identified to the species level. Samples from 2 animals (*Hylomyscus* sp and *Crocidura hildegardeae*) were positive for anti-OPXV IgG antibodies by ELISA. All small mammal samples were PCR negative for OPXV and MPXV DNA.

### Surveillance of Other Animal Groups

Two animals in other enclosures were temporarily quarantined during the outbreak. On 28 September, a male juvenile chimpanzee developed unilateral periorbital swelling but no other signs compatible with mpox. The swelling resolved with antihistamine and anti-inflammatory treatment and was considered allergic or traumatic in origin. On 2 October, an adult female crowned guenon monkey (*Cercopithecus pogonias*) escaped from her enclosure and came into proximity with the affected group, moving in areas contaminated with food waste and bodily excretions. She was quarantined for 3 weeks but developed no signs consistent with mpox.

On 28 August, an adult female chimpanzee in an unaffected group died suddenly. Gross necropsy findings were not consistent with mpox, and oropharyngeal and rectal swabs did not amplify MPXV DNA signatures by PCR.

### Control Measures

On 16 August, veterinary and public health authorities were informed. The sanctuary was closed to the public from 14 August and remained closed until the authorities permitted unaffected areas to reopen on 22 October. The entire park was reopened on 31 October.

An information dissemination session was conducted with all sanctuary staff on 19 August. The affected group was quarantined. The number of people in contact with the group was restricted to 2 keepers (exclusively dedicated to this group), 2 veterinarians, and 2 managers. Training in the use of personal protective equipment (PPE) was provided for these people, and dedicated clothing and cleaning equipment were used with this group. Proximity and direct contact with the affected group were restricted. Enhanced cleaning protocols were implemented, and a dedicated waste disposal pit was created within the quarantine zone.

Disinfection of the cages in the quarantined group was conducted every 7 to 9 days during the outbreak. Cleaning frequency was limited by practical factors, including animal cooperation, human health and safety, and restrictions on mixing animals imposed by dominance hierarchies within the group. A final disinfection was conducted on 24 October, when animals were no longer considered infectious.

### Human Surveillance

A contact was defined as anyone with direct contact with the affected group or its excretions, beginning 1 week prior to the onset of illness in the index case until the group was considered noninfectious for mpox. Contacts were identified through interviews with all sanctuary employees and volunteers. Contacts had daily temperature checks and self-monitored for rash development until 17 days from their last date of exposure. A total of 17 contacts were identified: 8 with confirmed direct contact, 7 self-reported contact with bodily fluids, and 2 self-reported unspecified contact. No individuals developed a fever or rash consistent with mpox.

## DISCUSSION

MPXV poses a risk to captive NHPs living in seminatural habitats in enzootic regions and, subsequently, human caregivers. Outbreaks have been reported in captive and wild NHPs, with variable attack rates of up to 100% and marked species differences [7, 16, 18, 31–33]. In this outbreak, the attack rate (87%) in chimpanzees was higher than typically reported in humans [30, 34–36]. This may be due to daily veterinary inspections enabling a high case identification rate [30] and/or differences in susceptibility, route of transmission, proximity, and behavior, with close physical contact and social grooming likely to increase transmission risk between NHPs [16]. Low attack rates in human outbreaks may also reflect cross-protection from smallpox vaccination [30].

There is a paucity of information on the clinical course, management, and epidemiology of natural MPXV infections in animals. Natural mpox outbreaks in NHPs could help to inform human care, particularly in low-resource settings [37]. We extrapolated data on incubation periods in human CI MPXV [30] to the onset date in the index case to estimate the earliest and latest likely exposure dates for a point source outbreak (Figure 1B, lines A and B). Thirteen cases (13/20, 65%) commenced >13 days (the upper quartile incubation period for human CI MPXV cases) after the latest likely exposure date for a point source outbreak (Figure 1B, line C). Acquisition of MPXV is thought to occur predominantly via the mucosal and percutaneous routes [38]. The outbreak commenced in subgroup A, in which access to a forested enclosure (Supplementary Figure 1) provides extensive opportunities to interact with potential reservoir species. This group has been seen to hunt wildlife. Infection was most likely via direct mucosal or percutaneous exposure to an infected reservoir host or fomites from a reservoir host by 1 or more of the chimpanzees. Additional animals were likely infected by direct chimp-to-chimp transmission or exposure to fomites. The delay in the onset of cases after the cessation of percutaneous and mucosal direct contact between the subgroups suggests that subgroup B animals, who did not have access to the forested enclosure and were confined to cages throughout, were likely indirectly infected by fomites or the respiratory route rather than via direct transmission. Although the cages were separated by 5 m, the chimpanzees could have thrown contaminated materials beyond this distance, and transfer of fomites by caregivers was also possible.

Experimental models [31] and observational studies [39] have demonstrated that the incubation period, prodrome, clinical signs, and severity of MPXV differ between host species and are dependent on the route and dose of infection. It has also been speculated that differences in clinical signs in wild chimpanzees infected with CII MPXV may reflect different routes of infection [16]. Different clinical syndromes were seen in this outbreak, with respiratory signs confined to subgroup A and a gastrointestinal syndrome observed in subgroup B. This may reflect different routes of transmission in the subgroups (supplementary materials).

Identifying early clinical signs that are predictive of increased morbidity or mortality could help to identify high-risk patients, optimize resource allocation, and improve treatment outcomes [37]. In this outbreak, edematous swelling was often the first localizing sign seen, occurring prior to or concurrent with exanthema. Facial and cervical swelling are common signs of mpox in a broad range of experimentally and naturally infected species, including NHPs [31, 40], but have not been reported in apes [16, 33, 41] and rarely in humans [10, 39, 42], except when associated with secondary bacterial infections [43]. Perilaryngeal edema is rarely referenced but may sometimes be a component of cervical swelling [31]. In this outbreak, perilaryngeal edema was predominantly found in subgroup A (7/13 [54%] in subgroup A vs 1/7 [14%] in subgroup B), which may suggest an association with the route of infection, potentially with a higher occurrence in animals exposed via the oropharyngeal route during ingestion of an infected reservoir host. Perilaryngeal edema was associated with more severe respiratory signs, present in all 5 chimpanzees that developed dyspnea and likely contributing to airway obstruction and death by asphyxiation in at least 1 animal. Anesthesia likely provoked or hastened death in that animal, particularly as the laryngeal swelling prevented placement of an endotracheal tube. There are occasional prior reports consistent with mpox-associated perilaryngeal edema leading to severe morbidity or mortality in humans and NHPs [10, 31, 33]. Severe respiratory signs were also a key feature of MPXV infections in wild chimpanzees [16]. If anesthesia of dyspneic mpox cases in any species is unavoidable, we recommend maintaining the patient in a prone position and providing airway support and supplemental oxygen.

Lymphadenopathy is commonly reported in natural and experimental mpox infections [31, 40, 44], occurring in the majority of CI MPXV cases in humans, typically prior to or concomitant with exanthema [2, 45]. Marked lymphadenopathy was seen in 3 of 4 chimpanzees experimentally infected with CII MPXV by the intravenous route [41] but was not reported in chimpanzees naturally infected with the same CII MPXV strain at Rotterdam Zoo [33]. Lymphadenopathy was observed in only 2 animals (10%) in this outbreak and was absent from all 4 animals examined under anesthesia or postmortem, suggesting that the low rate was not simply a result of low sensitivity of detection in conscious animals. Previous publications have suggested that differences in the immune response [10] or the route of infection [39] may affect the incidence of lymphadenopathy. Eschar is not a common sign of mpox but was seen in 35% of cases in this outbreak.

Phylogenetic analyses of the 2 available MPXV genomes from Cameroon—the 1989 human case from Ékoumdouma and the genome from this outbreak—suggest they share a most recent common ancestor. Limited bootstrap support (Supplementary Figure 4) indicates uncertainty of the relationships among several clusters of genomes, consistent with previous phylogeographic analyses of CI MPXV [46, 47]. Another study noted mutational differences within epidemiologically related clusters of cases [48]. Additional genomes from the region may provide insights into the phylogeography of MPXV within Cameroon and other areas of endemism.

Small mammal sampling in the sanctuary identified 2 small mammals (*Hylomyscus* sp and *Crocidura* sp) positive for anti-OPXV IgG antibodies by ELISA. The small mammal species sampled during this study contained only a few taxa that have been associated with MPXV in previous ecologic surveys. Despite research teams observing several squirrels of the genera *Heliosciurus* and *Funisciurus* at locations around the sanctuary, capture attempts were unsuccessful. Future studies and outbreak investigations should explore ways to humanely capture these taxa for sampling.

The detection of viable virus in samples collected from chimpanzees and parts of the chimpanzee enclosures accessible to human caregivers highlights the transmission risk to other animals and humans, given that viable MPXV can persist in certain environments [49]. Enclosures should be decontaminated frequently, and staff should utilize PPE when touching and cleaning contaminated surfaces. Routine use of PPE is not the norm in many sanctuaries in mpox-endemic countries due to limited financial resources, technical expertise, and supplies. Rapid risk assessment to identify situations in which PPE should be prioritized may reduce the risk of zoonotic spillover in sanctuaries (Supplementary Figure 5). Rapid diagnosis and early implementation of control measures, following a One Health approach, were likely important in preventing transmission to animals in other enclosures and humans.

## CONCLUSION

Mpox should be considered a differential diagnosis for facial swelling, perilaryngeal swelling, and eschar in chimpanzees in endemic areas, even in the absence of exanthema and especially where ≥2 animals are concurrently affected. In contrast to humans, lymphadenopathy may be insignificant or absent. Rapid identification of outbreaks reduces the risks to animals and humans, including animal caregivers at higher risk of exposure. Control measures should follow a One Health approach and include quarantining affected animals, limiting human contacts to essential care staff, surveillance of human contacts, use of PPE, and regular disinfection of enclosures. Vaccination of captive NHPs in endemic regions could also be considered.

## Supplementary Material

Supplementary text

Supplementary Table 1

Supplementary Table 2

Supplementary Table 3

Supplementary Table 4

Supplementary Table 5

Supplementary Figure 1

Supplementary Figure 2

Supplementary Figure 3

Supplementary Figure 4

Supplementary Figure 5

Supplementary Video

## Figures and Tables

**Table 1.** Frequency and Duration of Clinical Signs Observed in Symptomatic Animals (N = 20)

		Days, Median (Range)	
Clinical Sign	Animals Affected, No. (%)	Survivors	Fatalities
Any signs	20 (100)	28 (5–77)	8 (2–13)
Death	2 (10)	NA	8 (2–13)^a^
Prodrome^b^	15 (75)	3 (1–6)	5 (2–8)
Skin lesions	19 (95)	22 (5–56)	3 (1–5)
Exanthema^c^	18 (90)	16 (5–33)	3 (1–5)
Abscess/ulceration	8 (40)	25 (9–38)	NA
Eschar	7 (35)	23 (11–53)	1 (1)
Edema	14 (70)	6 (1–8)	5 (1–9)
Perilaryngeal	8 (40)	6 (1–7)	5 (1–9)
Facial	8 (40)	4 (2–8) ^d^	NA
Lethargy	17 (85)	4 (1–15)	7 (1–12)
Inappetence^e^	4 (20)	9 (2–15)	NA
Dysphagia	9 (45)	3 (1–6)	4 (1–7)
Respiratory signs^e^	7 (35)	4 (1–7)	3 (1–5)
Cough	4 (20)	2 (1–4)	4 (4)
Coryza^f^	5 (25)	4 (3–4)	3 (1–5)
Dyspnea^f^	5 (25)	5 (1–6)	1 (1)
Ocular signs^g^	3 (15)	65 (7–72) ^g^	NA
Lymphadenopathy^h^	2 (10)	NR	NA
Diarrhea	2 (10)	6 (2–9)	NA
Weight loss^i^	2 (10)	NR	NA
Medications^j^	18 (90)	12 (2–77)	7 (1–12)

Abbreviations: NA, not applicable; NR, not recorded.

a Duration of clinical signs prior to death. Anesthesia of the index case likely hastened death.

b Interval between the appearance of clinical signs and the development of exanthema. In 5 cases, no prodrome was observed. Of these, 3 showed exanthema on the first day that they were noted to be symptomatic. In the other 2, exanthema was not observed, but both animals matched the probable case definition. One of them was wary of humans so difficult to inspect. It is possible that exanthema was present but not observed. The other animal had 2 bleeding lesions on his face, which scabbed over. These may have been excoriated vesicles.

c Exanthema was defined as macular, papular, or pustular skin lesions. Lesion density varied among animals from single or sparsely distributed lesions to localized constellations or diffuse rash.

d One animal had a second episode of facial swelling, which coincided with a right postauricular abscess, 2 weeks after the first episode of facial edema had resolved. The timing, appearance, and suspected etiology of this swelling were markedly different from the facial edema seen early in the course of clinical signs, so it has been included in the statistics for abscess/ulceration.

e There were marked differences in clinical signs between the subgroups. Notably, respiratory signs were seen in 54% (95% CI, 26%–80%) of the affected animals in subgroup A but none of the animals in subgroup B (95% CI, 0%–44%; Fisher exact test, *P* = .044). Inappetence was noted in 57% (95% CI, 20%–88%) of the animals in subgroup B but no animals in subgroup A (95% CI, 0%–28%; Fisher exact test, *P* = .007). See Supplementary Table 2 for further details.

f Coryza and dyspnea were the only signs that showed borderline statistically significant associations with death, both present in 17% (95% CI, 4.4%–42%) of survivors and 100% of fatalities (95% CI, 20%–100%; Fisher exact test, *P* = .053).

g Ocular signs included blepharospasm, ocular discharge, corneal clouding, and corneal scarring. The duration of medically treated signs has been recorded.

h Affecting the cervical lymph nodes in one animal and the submandibular in the other. Both cases developed lymphadenitis.

i Marked, visually identified weight loss requiring dietary intervention.

j Duration that animals received medications, as prescribed by a veterinarian.
