# Supplementary material for: Clinical Manifestations of an Outbreak of Monkeypox Virus in Captive Chimpanzees in Cameroon, 2016
Source: J Infect Dis. Author manuscript; Available in PMC 2025 Mar 27. (PMC11949251; doi:10.1093/infdis/jiad601)
Supplement: Supplementary text [file NIHMS2060295-supplement-Supplementary_text.docx]

**SUPPLEMENTARY MATERIALS: CLINICAL MANIFESTATIONS OF AN OUTBREAK OF MONKEYPOX VIRUS IN CAPTIVE CHIMPANZEES IN CAMEROON, 2016**

| **CONTENTS** |  |
| --- | --- |
| **LIST OF ADDITIONAL SUPPLEMENTARY MATERIALS** | 2 |
| **SUPPLEMENTARY RESULTS** | 3 |
| Clinical Presentation | 3 |
| Clinical Treatment | 4 |
| **SUPPLEMENTARY DISCUSSION** | 6 |
| **REFERENCES** | 10 |

| **ADDITIONAL SUPPLEMENTARY MATERIALS**  N.B. not included in this document but accessible as individual files |
| --- |
| **SUPPLEMENTARY FIGURES** |
| Supplementary Figure 1: Schematic, non-scale diagram of the housing system for the affected group |
| Supplementary Figure 2: Mpox lesions in captive chimpanzees |
| Supplementary Figure 3: Histopathology of fatal MPXV in a chimpanzee |
| Supplementary Figure 4: Cladogram of the RAxML tree |
| Supplementary Figure 5: Zoonotic risk assessment flow chart |
| **SUPPLEMENTARY TABLES** |
| Supplementary Table 1: Environmental samples collected in and around the cages of affected chimpanzees |
| Supplementary Table 2: Frequency of clinical signs observed in symptomatic animals (n=20) by subgroup, with p-values for difference in proportions between the subgroups |
| Supplementary Table 3: Results for CI MPXV PCR assays and viral titration in cell culture from samples received and tested at CDC Atlanta |
| Supplementary Table 4: Small mammals sampled at Mefou Primate Sanctuary August 2016 and results from PCRs for OPXV and MPXV |
| Supplementary Table 5: Small mammals sampled at Mefou Primate Sanctuary December 2017 and results from anti-orthopox IgG ELISA, and PCRs for OPXV and MPXV |
| **SUPPLEMENTARY VIDEO** |
| Supplementary Video 1: Dyspnea and open mouth breathing in the index case |

## SUPPLEMENTARY RESULTS

### Clinical Presentation

Facial and peri-laryngeal swelling commenced in the prodromal period before the rash or concomitant with rash development. Facial swelling was usually unilateral (6/8, 75%), except in 2 animals with bilateral swelling of the supraorbital ridge. The periorbital region was frequently affected (5/8, 63%) and the extent of the swelling ranged from localized (4/8, 50%), to more extensive (4/8, 50%) e.g., post-auricular to submandibular. Concurrent lymphadenopathy was not observed but the distribution of swelling sometimes corresponded to lymph node chains in the head and neck (2/8, 25%). Peri-laryngeal swelling was bilaterally symmetrical and often accompanied by dysphagia (7/8, 88%) and/or dyspnea (5/8, 63%). Lymphadenopathy may have been a component of peri-laryngeal swelling but was not explicitly observed.

Eschar commenced concomitant with or after rash development and was usually located on the dorsum or face. Skin ulceration and/or abscessation of the face, dorsum or feet were observed in 35% of animals a median of 7 days (range 4-25 days) after the appearance of exanthema and were likely caused by secondary bacterial infections of the skin. Two animals (10%) developed post-auricular abscesses, in one case accompanied by pinnal necrosis. Two animals (10%) developed lymphadenitis. Two animals (10%) in subgroup B had dysenteric diarrhea leading to severe dehydration and required anesthetizing for intravenous fluid therapy. Two animals (10%) in subgroup B showed dramatic weight loss.

There were notable differences in clinical signs between the subgroups. Respiratory signs were seen in 54% (95% CI 26-80%) of the affected animals in subgroup A but none of the animals in subgroup B (95% CI 0-44%; Fisher’s exact test, p = 0.044). Respiratory signs included cough, coryza, dyspnea, and open mouth breathing; and severity ranged from mild to fatal. Conversely, gastrointestinal signs comprising inappetence, with or without diarrhea and weight loss, were seen in 57% (95%CI 20-88%) of animals in subgroup B but none of the animals in subgroup A (95% CI 0-28%; Fisher’s exact test, p = 0.007). For individual clinical signs (except inappetence), differences between the subgroups did not approach statistical significance, which may represent a type II error due to the low number of animals in each group.

### Clinical Treatment

Medications were administered orally unless this was not possible for health and safety reasons, animals did not comply, or animals required parenteral treatment due to the severity of illness. Compliance was impacted by individual animal temperament, severity of illness, and the group dominance hierarchy.

Drug doses were based on estimated body weights. All animals were given daily multivitamin tablets during the outbreak and dewormed once with 400mg albendazole. If sedation was deemed necessary for veterinary examination and treatment purposes, animals were immobilized with either 3mg/kg ketamine and 0.03mg/kg medetomidine, or 5-10mg/kg ketamine with or without 0.25mg/kg diazepam.

Peri-laryngeal swelling and dysphagia were treated with corticosteroids: 20-40mg prednisolone once (SID) or twice (BID) daily dependent on severity. Intramuscular dexamethasone injections (4mg SID to every other day) were used in animals that refused oral medications. Analgesia was managed using acetaminophen 500-600mg BID and a slow-release formulation of tramadol 100-150mg BID. Of eight animals with peri-laryngeal edema, seven were treated with corticosteroids and tramadol (the remaining animal, the index case, died prior to diagnosis of mpox and before treatment could be administered). In 3/7 (43%) animals treated with corticosteroids and tramadol, the swelling visibly reduced and demeanor improved within 2-24h of administration. Of the four remaining animals, one improved within 24h of increasing the dose of corticosteroid (within 48h of initial presentation). The remaining three animals showed poor compliance with medication, refusing some doses, particularly within the first 24h of presentation. One of these three improved within 24h of increasing the dose of corticosteroid (within 48h of initial presentation), one improved over several days, and the remaining animal, the second fatality, continued to show poor compliance up until her death.

Non-steroidal anti-inflammatories (naproxen 220mg BID or ibuprofen 400mg BID to three times a day (TID)) were used in animals that were not receiving corticosteroids if they were lethargic, inappetent or had inflammatory skin lesions. Animals with open or discharging skin lesions were given antimicrobials, predominantly co-amoxiclav 500-625mg BID, but oxytetracycline 500-750mg BID as an alternative in animals that refused co-amoxiclav. If animals complied, discharging wounds were cleaned or flushed with povidone-iodine or chlorhexidine. Diarrhea was managed with fluid support and antimicrobials (metronidazole and oxytetracycline). Carbocisteine 150mg BID was used as an expectorant in animals with a cough. It was not possible to give topical ocular medications due to lack of compliance. A course of oral acyclovir (200mg TID x 8 days) was given in one animal with ocular involvement. This animal’s ocular signs subsequently appeared to fully resolve with no apparent chronic effects. Animals with marked weight loss were given dietary caloric and protein supplementation.

## SUPPLEMENTARY DISCUSSION

In wild chimpanzees naturally infected with CII MPXV, a respiratory syndrome with limited to absent exanthema has been described [1]. This study demonstrates a similar syndrome occurring in some captive chimpanzees naturally infected with CI MPXV. This respiratory syndrome was seen exclusively in animals in subgroup A. Signs included cough, coryza, oral discharge, dyspnea, and open mouth breathing; severity ranged from mild to fatal. We noted that peri-laryngeal swelling was present in all 5 animals that developed dyspnea, suggesting that edema can make an important contribution to the severity of respiratory signs in chimpanzees with mpox. Additionally, we found that extensive exanthema was not a reliable feature of mpox in captive chimpanzees, correlating with similar observations in wild chimpanzees [1]. Exanthema was absent (or unobserved) in some animals that matched the case definition for mpox. Where exanthema was present, most animals showed individual or sparse, circumscribed lesions, whilst some developed localized constellations or diffuse rash. Localized or diffuse rash with a high number of lesions appeared to be associated with more severe clinical signs, particularly respiratory signs, and was observed in both fatalities.

Corticosteroids were empirically prescribed in an attempt to reduce peri-laryngeal edema and decrease the risk of resultant airway obstruction. This aligns with a human case report that describes an mpox patient with an increasingly sore throat leading to dyspnea and a sense of airway obstruction, which improved on treatment with corticosteroids [2].The efficacy of this treatment in chimpanzees with peri-laryngeal edema was unclear, as although 5/7 (71%) improved within 48h of treatment (3 of these within 24h), there was no control group for comparison, and 3/7 animals (including the two that did not improve within 48h) showed poor compliance with taking medications, with one animal ultimately dying.

Differences in clinical signs between the subgroups may reflect different routes of transmission. Respiratory signs were only seen in subgroup A, whilst inappetence, diarrhea and weight loss, were only seen in subgroup B. Both fatalities were in subgroup A. A review of experimental studies in NHPs [3] reported that percutaneous challenge, particularly with CI MPXV, typically caused severe disease, with one study noting prominent inappetence, diarrhea, and weight loss [4], similar to the signs seen in some animals in subgroup B. Experimental infection via the respiratory tract typically led to bronchopneumonia and high mortality, although this was less apparent with intranasal infection where dose volume had a marked effect on the pathogenesis [3]. With low dose volume, the infection remained localized to the upper respiratory tract, whereas larger volumes resulted in spread to the lower respiratory tract. A study of human infections with CII MPX in the US 2003 outbreak divided cases based on potential route of exposure [5]. If an invasive exposure (e.g., a bite or scratch sufficient to break the skin) was reported, this was classified as a ‘complex’ exposure, otherwise the exposure was classified as ‘non-invasive’. It was recognized that in both groups, non-invasive (e.g., respiratory or mucosal) transmission may have occurred. Complex exposures were associated with shorter incubation periods, increased systemic signs, and more severe illness. Reviewing our findings and previous studies, no consistent picture emerges of particular transmission routes leading to typical clinical signs and courses of infection. Unravelling the roles of pathogen factors, transmission route, infectious dose, and host susceptibility in the epidemiology and pathogenesis of MPXV present an ongoing challenge, which experimental data and field studies have, as yet, failed to resolve. However, there is certainly a suggestion that route of transmission may affect the clinical presentation, and this was also postulated to play a role in clinical differences in outbreaks in wild chimpanzees [1]. This should remain an area of focus for future experimental studies, as well as observational studies in humans and animals.

References

1. Patrono LV, Pleh K, Samuni L, et al. Monkeypox virus emergence in wild chimpanzees reveals distinct clinical outcomes and viral diversity. Nature Microbiology **2020**; 5:955-+.

2. Sejvar JJ, Chowdary Y, Schomogyi M, et al. Human monkeypox infection: a family cluster in the midwestern United States. The Journal of infectious diseases **2004**; 190:1833-40.

3. Parker S, Buller RM. A review of experimental and natural infections of animals with monkeypox virus between 1958 and 2012. Future virology **2013**; 8:129-57.

4. Saijo M, Ami Y, Suzaki Y, et al. Virulence and pathophysiology of the Congo Basin and West African strains of monkeypox virus in non-human primates. Journal of General Virology **2009**; 90:2266-71.

5. Reynolds MG, Yorita KL, Kuehnert MJ, et al. Clinical manifestations of human Monkeypox influenced by route of infection. The Journal of infectious diseases **2006**; 194:773-80.
