# Supplementary material for: Clinical Manifestations of an Outbreak of Monkeypox Virus in Captive Chimpanzees in Cameroon, 2016
Source: J Infect Dis. Author manuscript; Available in PMC 2025 Mar 27. (PMC11949251; doi:10.1093/infdis/jiad601)
Supplement: Supplementary Table 1 [file NIHMS2060295-supplement-Supplementary_Table_1.docx]

**Supplementary Table 1.** Environmental samples collected in and around the cages of affected chimpanzees.

| **Samples collected** | **Type** | **Number of pairs** |
| --- | --- | --- |
| Floor | Swab | 2 |
| Exterior bars | Swab | 1 |
| Internal separation bars | Swab | 1 |
| Sleeping platform | Swab | 2 |
| Sleeping platform (underside) | Swab | 1 |
| Padlock | Swab | 1 |
| Floor bedding | Swab | 1 |
| Chewed sugar cane | Fibers | 1 |
| Bedding material (plants) | Plant material | 1 |
| Soil from outside cage | Soil | 1 |
| Feces | Feces | 1 |
| **Total** |  | **13** |
