# Supplementary material for: Clinical Manifestations of an Outbreak of Monkeypox Virus in Captive Chimpanzees in Cameroon, 2016
Source: J Infect Dis. Author manuscript; Available in PMC 2025 Mar 27. (PMC11949251; doi:10.1093/infdis/jiad601)
Supplement: Supplementary Table 2 [file NIHMS2060295-supplement-Supplementary_Table_2.docx]

**Supplementary Table 2.** Frequency of clinical signs observed in symptomatic animals (n=20) by subgroup, with p-values for difference in proportions between the subgroups.

| CLINICAL SIGN | ANIMALS AFFECTED WITH 95% CONFIDENCE INTERVALS  n (%) | | | | |  |
| --- | --- | --- | --- | --- | --- | --- |
|  | Total  (n=20) | Subgroup A (n=13) | 95% CI | Subgroup B (n=7) | 95% CI | p-value |
| Attack rate^a^ | 20/23^a^ (87) | 13/16a (81) | 54-95 | 7/7^a^ (100) | 56-100 | 0.5 |
| Death | 2 (10) | 2 (15) | 3-46 | 0 (0) | 0-44 | 0.5 |
| Prodrome | 15 (75) | 10 (77) | 46-94 | 5 (71) | 30-95 | >0.9 |
| Skin lesions: | 19 (95) | 13 (100) | 72-100 | 6 (86) | 42-99 | 0.4 |
| Exanthema | 18 (90) | 13 (100) | 72-100 | 5 (71) | 30-95 | 0.11 |
| Abscess/ulceration | 8 (40) | 5 (38) | 15-68 | 3 (43) | 12-80 | >0.9 |
| Eschar | 7 (35) | 3 (23) | 6-54 | 4 (57) | 20-88 | 0.2 |
| Edema: | 14 (70) | 9 (69) | 39-90 | 5 (71) | 30-95 | >0.9 |
| Peri-laryngeal | 8 (40) | 7 (54) | 26-80 | 1 (14) | 1-58 | 0.2 |
| Facial | 8 (40) | 4 (31) | 10-61 | 4 (57) | 20-88 | 0.4 |
| Respiratory signs: | 7 (35) | 7 (54) | 26-80 | 0 (0) | 0-44 | **0.04** |
| Cough | 4 (20) | 4 (31) | 10-61 | 0 (0) | 0-44 | 0.2 |
| Coryza | 5 (25) | 5 (38) | 15-68 | 0 (0) | 0-44 | 0.11 |
| Dyspnea | 5 (25) | 5 (38) | 15-68 | 0 (0) | 0-44 | 0.11 |
| Lethargy | 17 (85) | 10 (77) | 46-94 | 7 (100) | 56-100 | 0.5 |
| Inappetence | 4 (20) | 0 (0) | 0-28 | 4 (57) | 20-88 | **0.007** |
| Dysphagia | 9 (45) | 7 (54) | 26-80 | 2 (29) | 5-70 | 0.4 |
| Ocular signs^b^ | 3 (15) | 1 (8) | 0-38 | 2 (29) | 5-70 | 0.3 |
| Lymphadenopathy^c^ | 2 (10) | 1 (8) | 0-38 | 1 (14) | 1-58 | >0.9 |
| Diarrhea | 2 (10) | 0 (0) | 0-28 | 2 (29) | 5-70 | 0.11 |
| Weight loss^d^ | 2 (10) | 0 (0) | 0-28 | 2 (29) | 5-70 | 0.11 |
| Medications^e^ | 18 (90) | 11 (85) | 54-97 | 7 (100) | 56-100 | 0.5 |

^a^ The denominator is total animals in the group (overall: n = 23, subgroup A: n = 16, subgroup B: n = 7).

^b^ Ocular signs included blepharospasm, ocular discharge, corneal clouding, and corneal scarring. The duration of medically treated signs has been recorded.

^c^ Affecting the cervical lymph nodes in one animal and the submandibular in the other. Both cases developed lymphadenitis.

^d^ Marked, visually identified weight loss requiring dietary intervention.

^e^ Duration animals received medications, as prescribed by a veterinarian.
