# Supplementary material for: Clinical Manifestations of an Outbreak of Monkeypox Virus in Captive Chimpanzees in Cameroon, 2016
Source: J Infect Dis. Author manuscript; Available in PMC 2025 Mar 27. (PMC11949251; doi:10.1093/infdis/jiad601)
Supplement: Supplementary Table 3 [file NIHMS2060295-supplement-Supplementary_Table_3.docx]

**Supplementary Table 3.** Results for CI MPXV PCR assays and viral titration in cell culture from samples received and tested at CDC Atlanta. NP indicates no plaques were observed in cell culture. Cycle threshold (CT) value <37 considered positive for MPXV.

| **SAMPLE DATE** | **SAMPLE SOURCE** | **SAMPLE TYPE** | **CI MPXV PCR***  **AVG CT** | **TITER**  **(PFU/ML)** |
| --- | --- | --- | --- | --- |
| 16/08/16 | Index case | Tongue | 18.9 | 6.54E+6 |
| 16/08/16 | Index case | Colon | 20.2 | 5.23E+6 |
| 16/08/16 | Index case | Lung | 22.9 | 7.46E+5 |
| 16/08/16 | Index case | Larynx | 23.6 | 5.15E+5 |
| 16/08/16 | Index case | Spleen | 23.2 | 3.62E+5 |
| 16/08/16 | Index case | Small Intestine | 23.3 | 7.69E+4 |
| 16/08/16 | Index case | Lymph Node | 25.0 | 3.38E+4 |
| 16/08/16 | Index case | Heart | 29.9 | 9.23E+1 |
| 16/08/16 | Index case | Kidney | 33.7 | 1.54E+1 |
| 16/08/16 | Index case | Skin lesion | 17.3 | cytotoxic |
| 16/08/16 | Index case | Stomach | 31.8 | cytotoxic |
| 16/08/16 | Index case | Liver | 32.6 | NP |
| 26/08/16 | Chewed sugar cane | Environmental Material | 29.2 | cytotoxic |
| 26/08/16 | Cage exterior bars | Environmental Swab | 36.6 | NP |
| 26/08/16 | Cage shared bars | Environmental Swab | 36.3 | NP |
| 26/08/16 | Top of sleeping platform | Environmental Swab | 31.8 | 5.00E+2 |
| 26/08/16 | Top of sleeping platform | Environmental Swab | 31.9 | 6.15E+1 |
| 26/08/16 | Bedding from ground | Environmental Swab | 29.6 | cytotoxic |
| 04/09/16 | Chimpanzee 2 | Oral Swab | 27.3 | cytotoxic |
| 04/09/16 | Chimpanzee 3 | Oral Swab | 30.0 | cytotoxic |

*PCR amplicons from positive samples were sequenced; all sequences obtained were identical 302 bp fragments which corresponded to Topoisomerase 1 OPXV sequences. They were also identical to sequences obtained from symptomatic animals from the same enclosure.
