# Supplementary material for: Clinical Manifestations of an Outbreak of Monkeypox Virus in Captive Chimpanzees in Cameroon, 2016
Source: J Infect Dis. Author manuscript; Available in PMC 2025 Mar 27. (PMC11949251; doi:10.1093/infdis/jiad601)
Supplement: Supplementary Table 4 [file NIHMS2060295-supplement-Supplementary_Table_4.docx]

**Supplementary Table 4.** Small mammals sampled at Mefou Primate Sanctuary August 2016 and results from PCRs for OPXV and MPXV.

| **Order** | **Genus** | **No. of animals sampled** | **No. of PCR positives** |
| --- | --- | --- | --- |
| Rodentia | Cricetomys | 1 | 0 |
| Rodentia | Rattus | 9 | 0 |
| Rodentia | Mus | 25 | 0 |
| Rodentia | Lophuromys | 14 | 0 |
| Rodentia | Praomys | 7 | 0 |
| Rodentia | Not identified | 14 | 0 |
| Eulipotyphia | Not identified | 7 | 0 |
| **Total** |  | **77** | **0** |
