# Supplementary material for: Clinical Manifestations of an Outbreak of Monkeypox Virus in Captive Chimpanzees in Cameroon, 2016
Source: J Infect Dis. Author manuscript; Available in PMC 2025 Mar 27. (PMC11949251; doi:10.1093/infdis/jiad601)
Supplement: Supplementary Table 5 [file NIHMS2060295-supplement-Supplementary_Table_5.docx]

**Supplementary Table 5.** Small mammals sampled at Mefou Primate Sanctuary December 2017 and results from anti-orthopox IgG ELISA, and PCRs for OPXV and MPXV.

| **Order** | **Genus** | **Animals sampled**  **(n)** | **ELISA positive**  **(n)** | **IgG ELISA positive**  **(%)** | **PCR positive**  **(n)** |
| --- | --- | --- | --- | --- | --- |
| Rodentia | Praomys | 47 | 0 | 0.0 | 0 |
| Rodentia | Hylomyscus | 6 | 1 | 16.7 | 0 |
| Rodentia | Cricetomys | 3 | 0 | 0.0 | 0 |
| Rodentia | Hybomys | 3 | 0 | 0.0 | 0 |
| Rodentia | Lophuromys | 3 | 0 | 0.0 | 0 |
| Rodentia | Mus | 2 | 0 | 0.0 | 0 |
| Rodentia | Deomys | 1 | 0 | 0.0 | 0 |
| Rodentia | Stochomys | 1 | 0 | 0.0 | 0 |
| Eulipotyphia | Crocidura | 13 | 1 | 7.7 | 0 |
| Eulipotyphia | Suncus | 5 | 0 | 0.0 | 0 |
| Eulipotyphia | Sylvisorex | 1 | 0 | 0.0 | 0 |
| **Total** |  | **85** | **2** | **2.4** | **0** |
