# Supplementary figures and images for: Clinical Manifestations of an Outbreak of Monkeypox Virus in Captive Chimpanzees in Cameroon, 2016
Source: J Infect Dis. Author manuscript; Available in PMC 2025 Mar 27. (PMC11949251; doi:10.1093/infdis/jiad601)

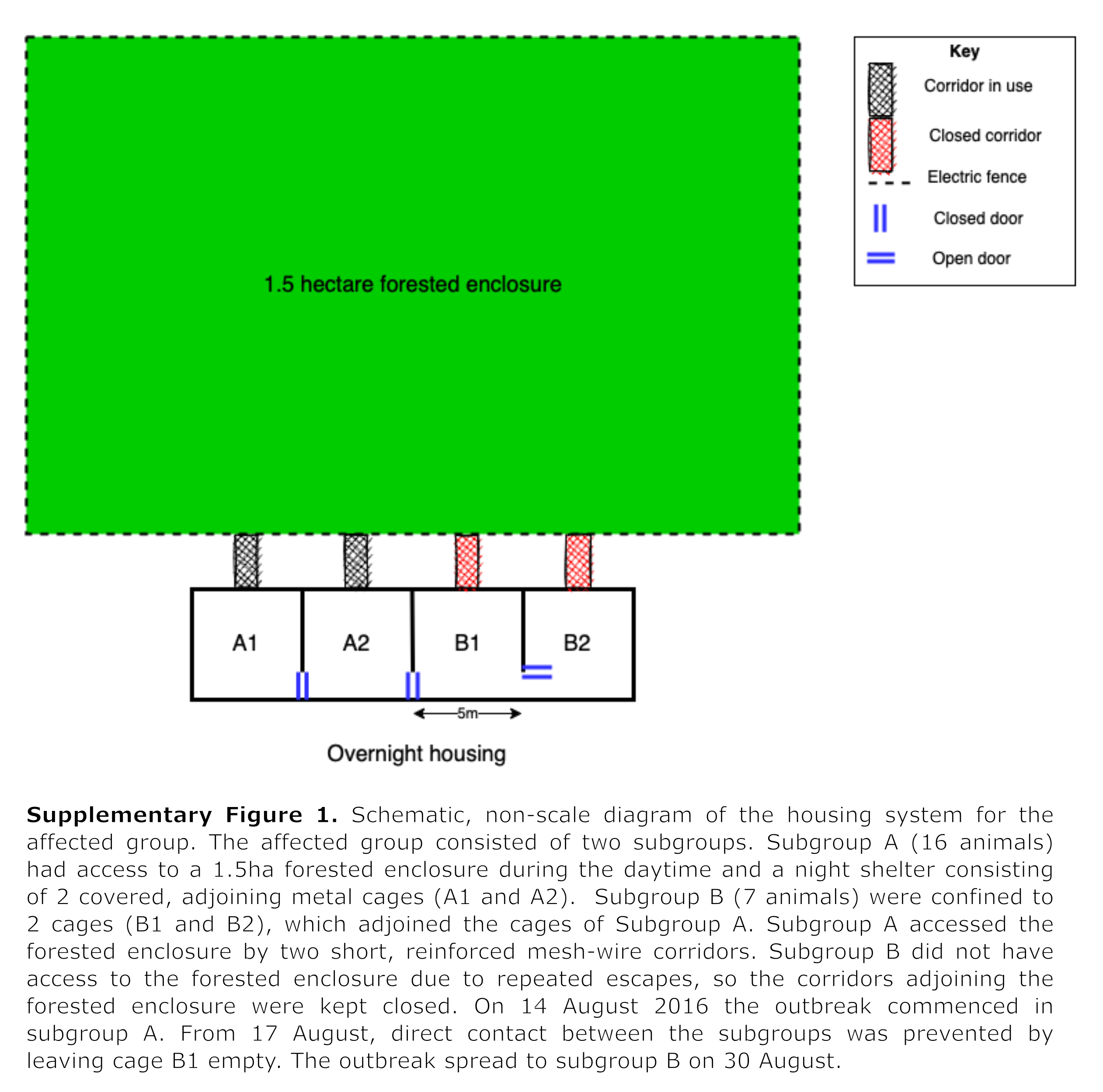

Supplement: Supplementary Figure 1 [file NIHMS2060295-supplement-Supplementary_Figure_1.png]

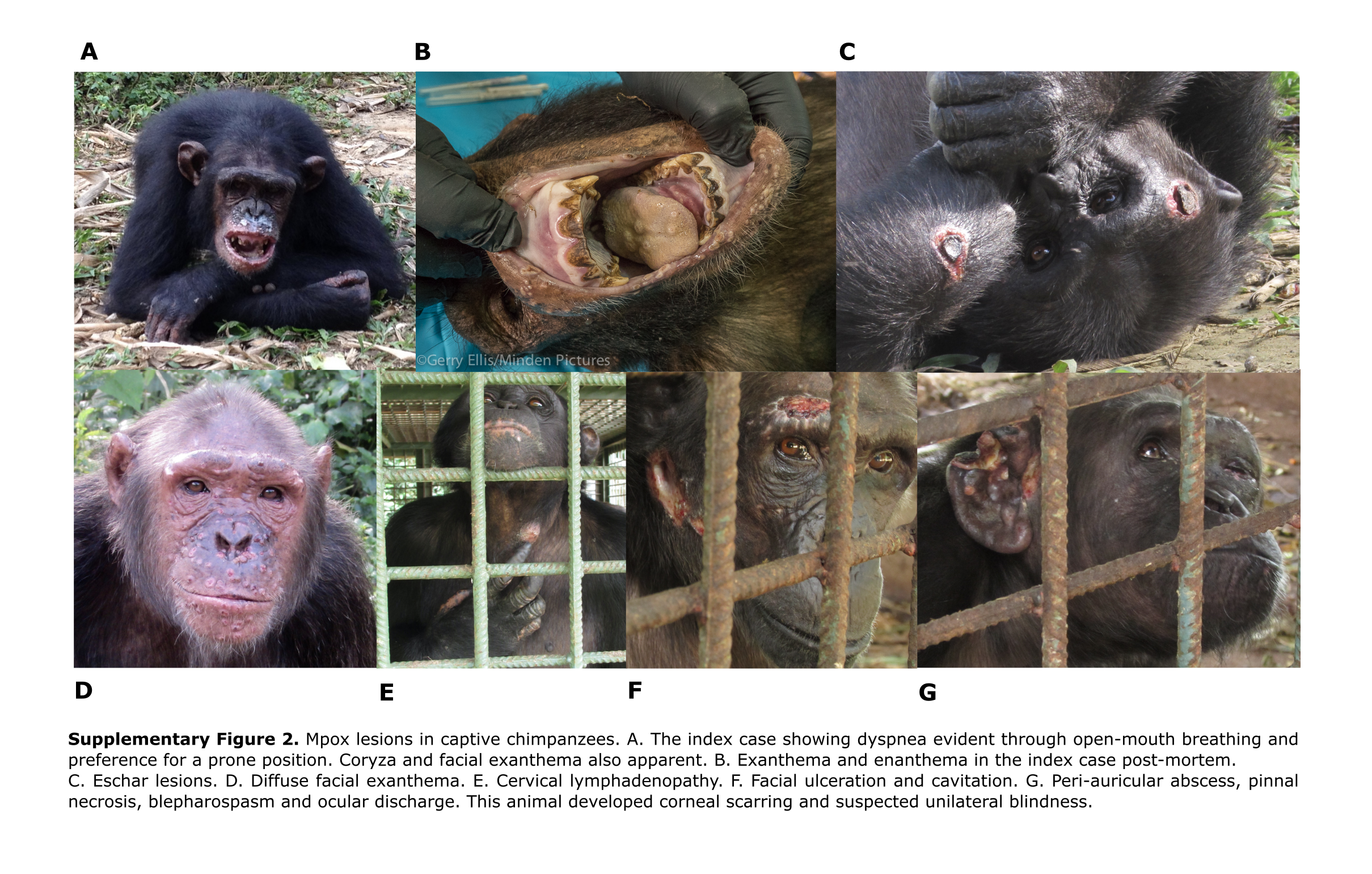

Supplement: Supplementary Figure 2 [file NIHMS2060295-supplement-Supplementary_Figure_2.png]

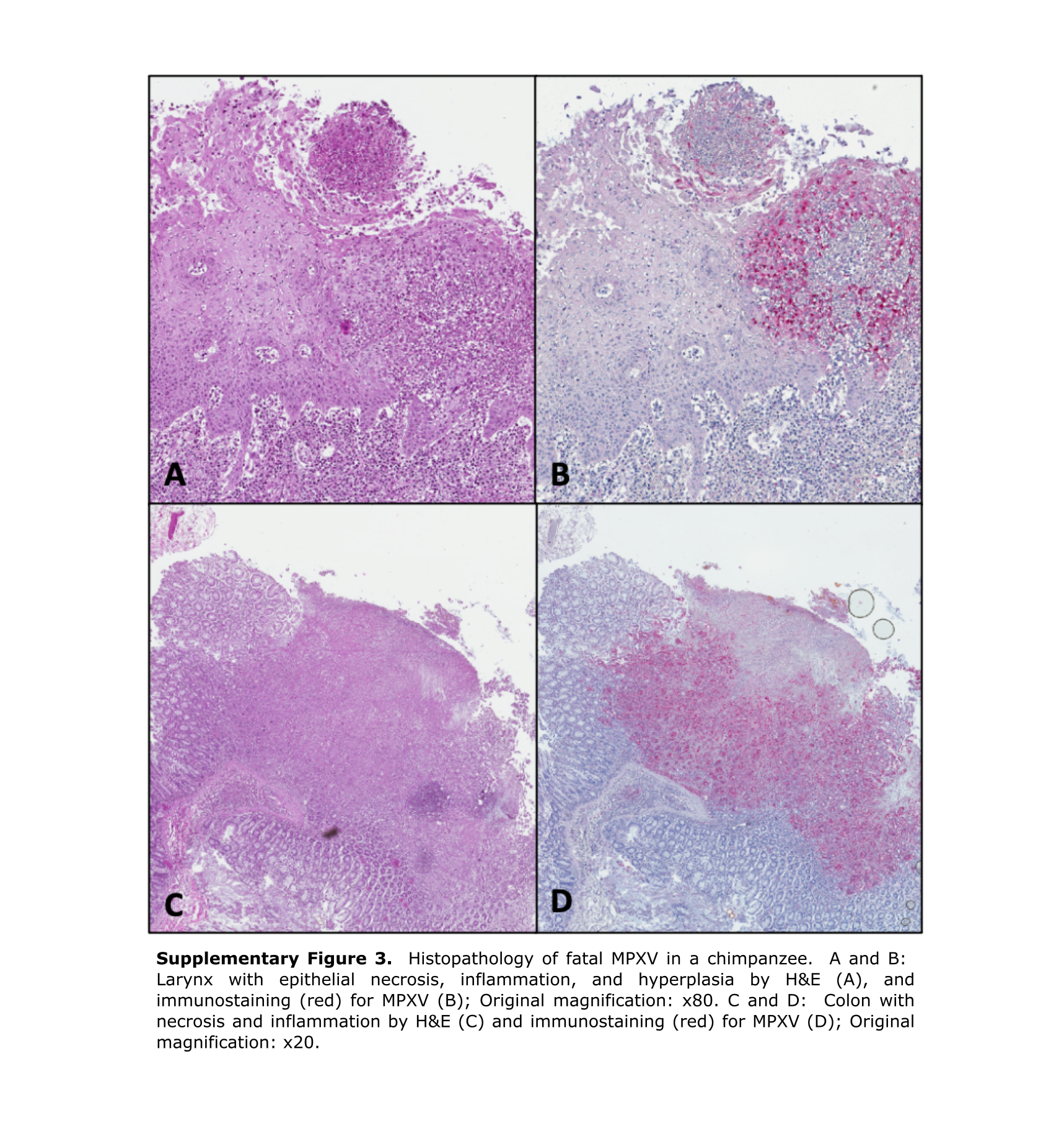

Supplement: Supplementary Figure 3 [file NIHMS2060295-supplement-Supplementary_Figure_3.png]

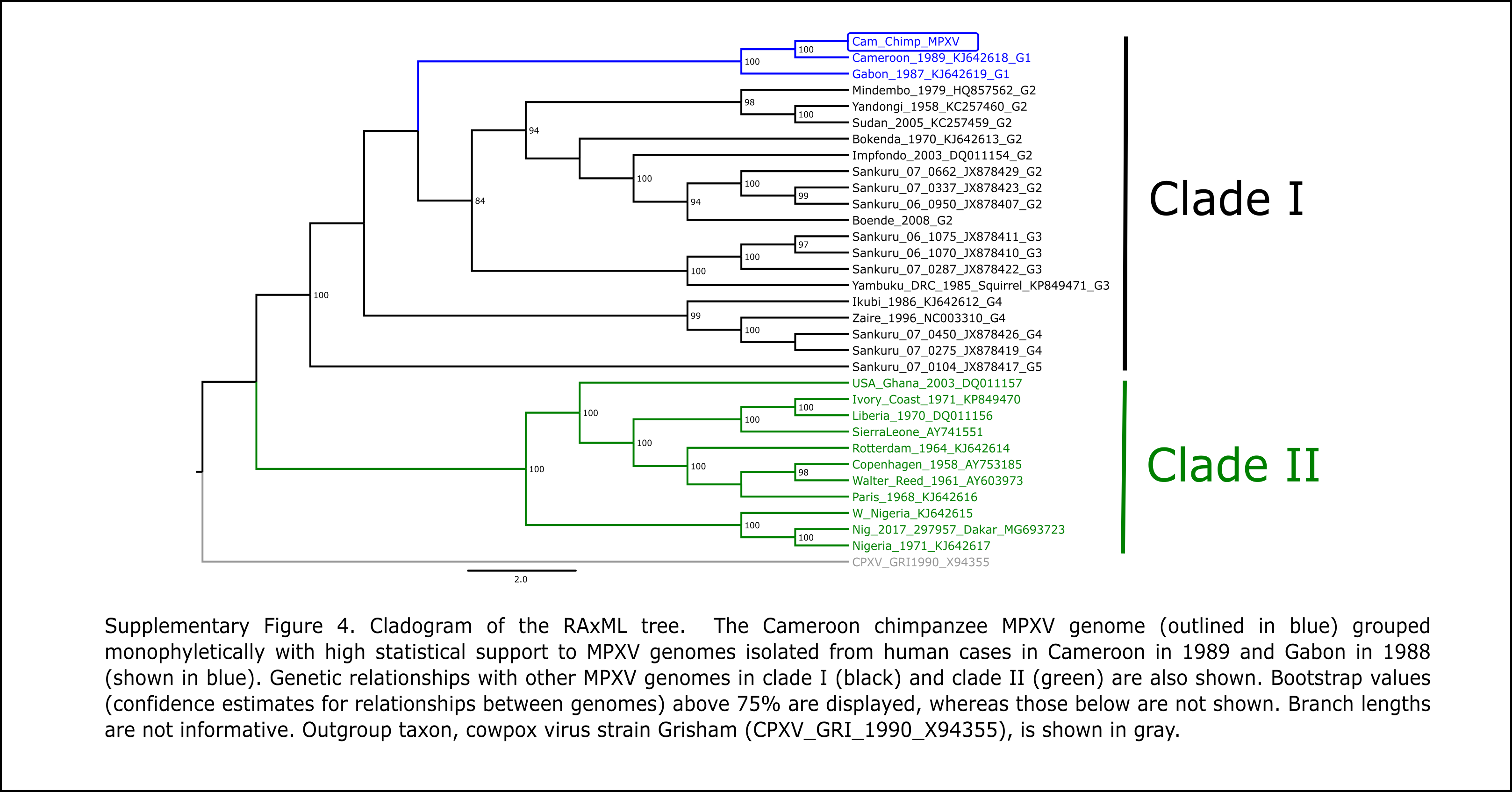

Supplement: Supplementary Figure 4 [file NIHMS2060295-supplement-Supplementary_Figure_4.png]

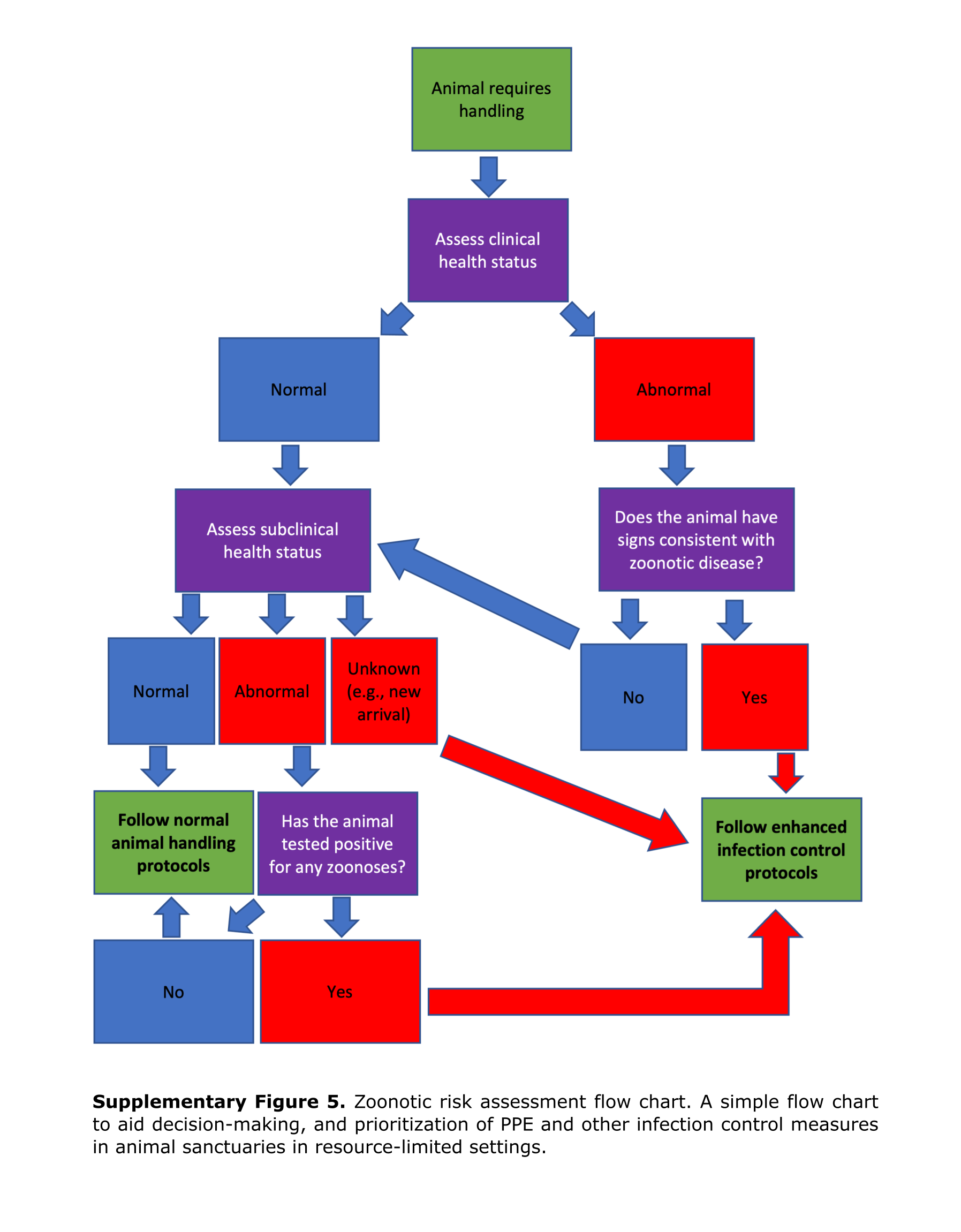

Supplement: Supplementary Figure 5 [file NIHMS2060295-supplement-Supplementary_Figure_5.png]
